# Successful management of a patient with ovarian ectopic pregnancy by the end of the first trimester: a case report

**DOI:** 10.1186/s13256-022-03403-w

**Published:** 2022-05-02

**Authors:** Sara Kasraei, Akram Seifollahi, Faezeh Aghajani, Amin Nakhostin-Ansari, Neda Zarei, Afsaneh Tehranian

**Affiliations:** 1 Department of Obstetrics and Gynecology, Arash Women’s Hospital, Tehran University of Medical Sciences, Rashid Ave, Resalat Highway, Tehranpars, Tehran, Iran; 2 Department of Pathology, Arash Women’s Hospital, Tehran University of Medical Sciences, Tehran, Iran; 3 Research Development Center, Arash Women’s Hospital, Tehran University of Medical Sciences, Tehran, Iran; 4 Sports Medicine Research Center, Neuroscience Institute, Tehran University of Medical Sciences, Tehran, Iran

**Keywords:** Ectopic pregnancy, Ovary

## Abstract

**Background:**

Among all ectopic pregnancies, between 0.5% and 3.5% are ovarian ectopic pregnancies, a potentially life-threatening condition when ruptured due to its serious potential for hemorrhaging. A majority of ovarian ectopic pregnancies are diagnosed by the 7th week of pregnancy when the patient becomes symptomatic, and ultrasound can be used to diagnose this condition.

**Case presentation:**

We present the case of a 39-year-old Persian woman in the 12th week of gestation who presented with vaginal bleeding and abdominal pain and was diagnosed with ovarian ectopic pregnancy. Her notable laboratory finding was β-human chorionic gonadotropin > 15,000, which indicates definite pregnancy. Transvaginal ultrasound (TVS) revealed no evidence of intrauterine pregnancy, but a well-circumscribed gestational sac in the left ovary. The patient was successfully treated with resection of the gestational sac and partial left salpingo-oophorectomy. Histopathological studies confirmed the diagnosis of ovarian ectopic pregnancy.

**Conclusion:**

The case emphasizes the ability of ovarian ectopic pregnancy to develop asymptomatically through the course of pregnancy and points to the necessity for high-quality prenatal care and the importance of determining the fetal site during pregnancy.

## Background

Ectopic pregnancy constitutes 1–2% of all pregnancies and is among the leading causes of maternal morbidity and mortality. Ovarian ectopic pregnancy (OEP) is one of the rarest subtypes, with an estimated incidence of 0.5–3.5% of all ectopic pregnancies, which is increasing in the past decades [1, 2]. In most cases, ovarian pregnancies terminate with rupture in the first trimester, which has potential for life-threatening massive internal hemorrhage [3]. OEP shares a similar clinical presentation with complicated ovarian cyst and tubal ectopic pregnancy [4], thus its preoperative diagnosis is challenging, and most cases of OEP are diagnosed intraoperatively [5]. The etiology of OEP is not fully understood, but it has been reported to be associated with utilizing an intrauterine device (IUD) in many cases [6]. We report a case of OEP with an accurate preoperative diagnosis by transvaginal ultrasound (TVS) with confirmation during laparotomy and histopathological examination.

## Case presentation

The patient was a 39-year-old pregnant Iranian woman, G4P3L3 (gravidity 4, parity 3, live births 3), who presented to the emergency department of a specialized women’s and neonatal hospital with spotting and abdominal pain at the 6th week of gestation based on the reported last menstrual period (LMP). In her obstetrician history, she had three pregnancies delivered by natural vaginal delivery (NVD) at term without any complications. She had a history of contraceptive IUD use for the last 3 years, which was removed 2 months before the current admission due to spotting. The patient did not mention any other symptoms. Her past medical history, drug history, and family history were otherwise unremarkable. The patient mentioned no history of smoking or drinking alcohol.

**Fig. 1:**
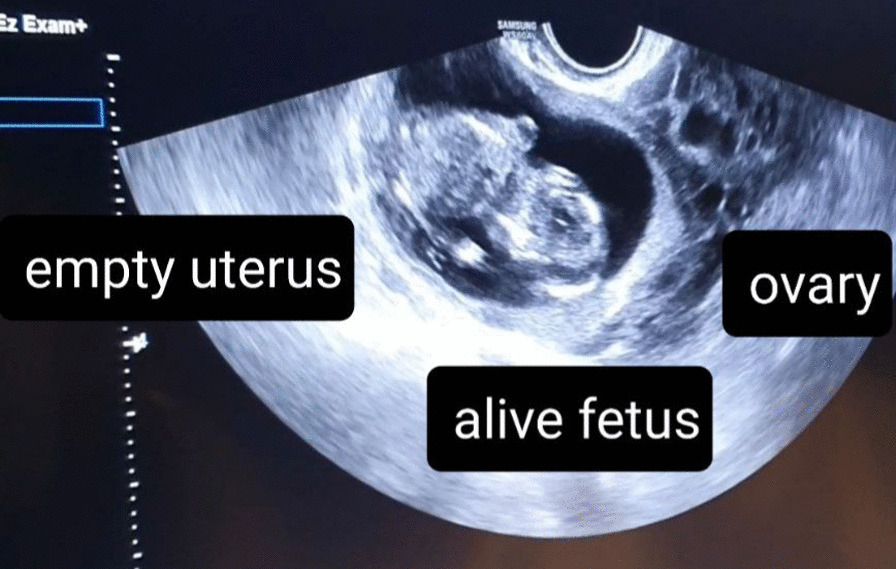
Evidence of ovarian ectopic pregnancy (EP) in the Transvaginal sonography (TVS)

On physical examination, her vital signs were in normal ranges. Her abdomen was firm, without tenderness, rebound tenderness, guarding, or rigidity. Otherwise, her physical examination was unremarkable. Her notable laboratory finding was β-human chorionic gonadotropin (HCG) > 15,000, which indicates definite pregnancy. Transvaginal ultrasound (TVS) showed no evidence of intrauterine pregnancy, but a well-circumscribed gestational sac in the left ovary with crown–rump length (CRL) of 55 mm, compatible with a gestational age of 12 weeks and 1 day and visible fetal heart rate (FHR) of the fetus, highly suggestive of left ovarian ectopic pregnancy (Fig. 1). TVS also revealed mild pelvic free fluid.

**Fig. 2:**
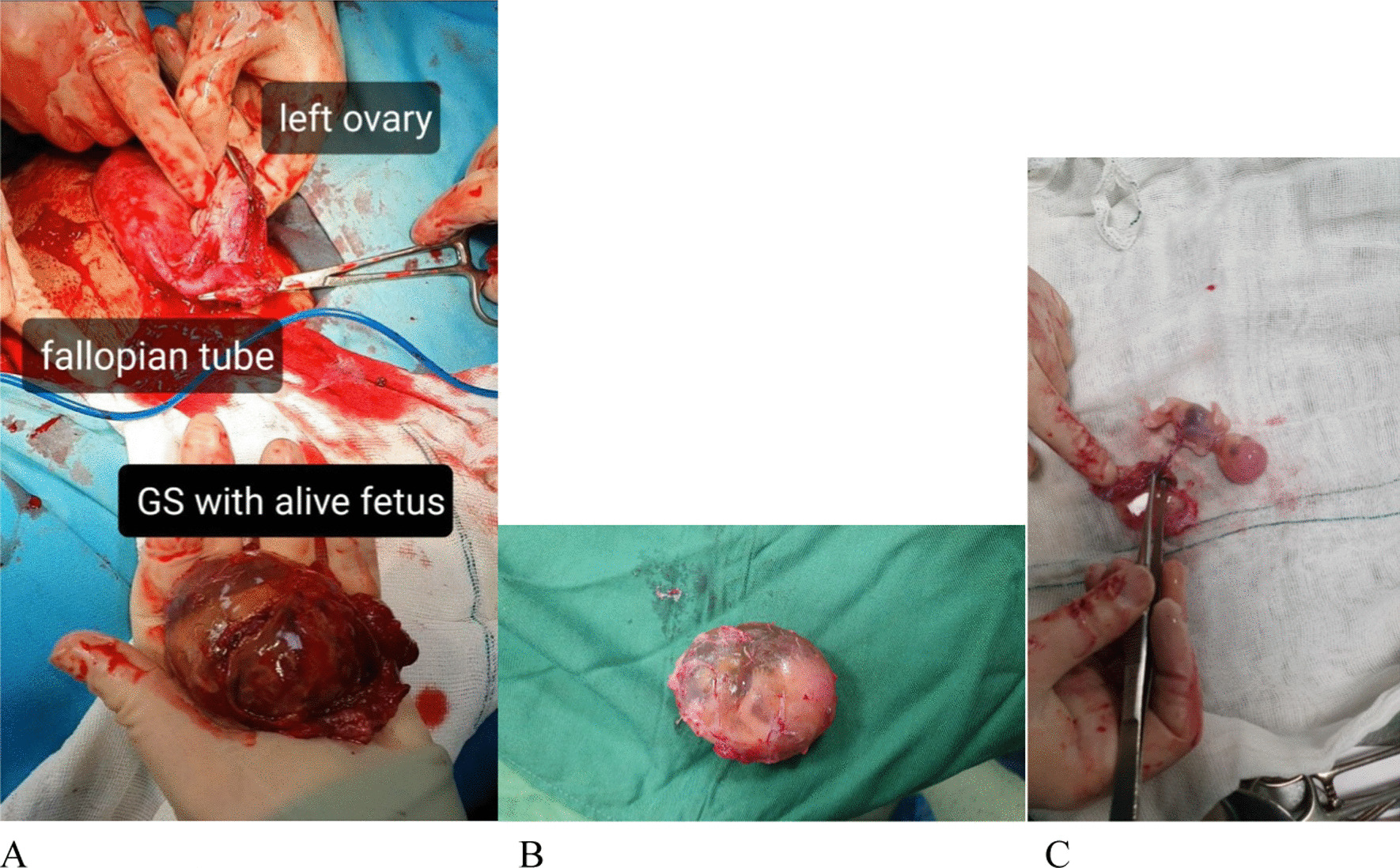
Intraoperative pictures. **A** Resection of the gestational sack from the ovary. **B** Gestational sack. **C** Fetus in the gestational sack

**Fig. 3:**
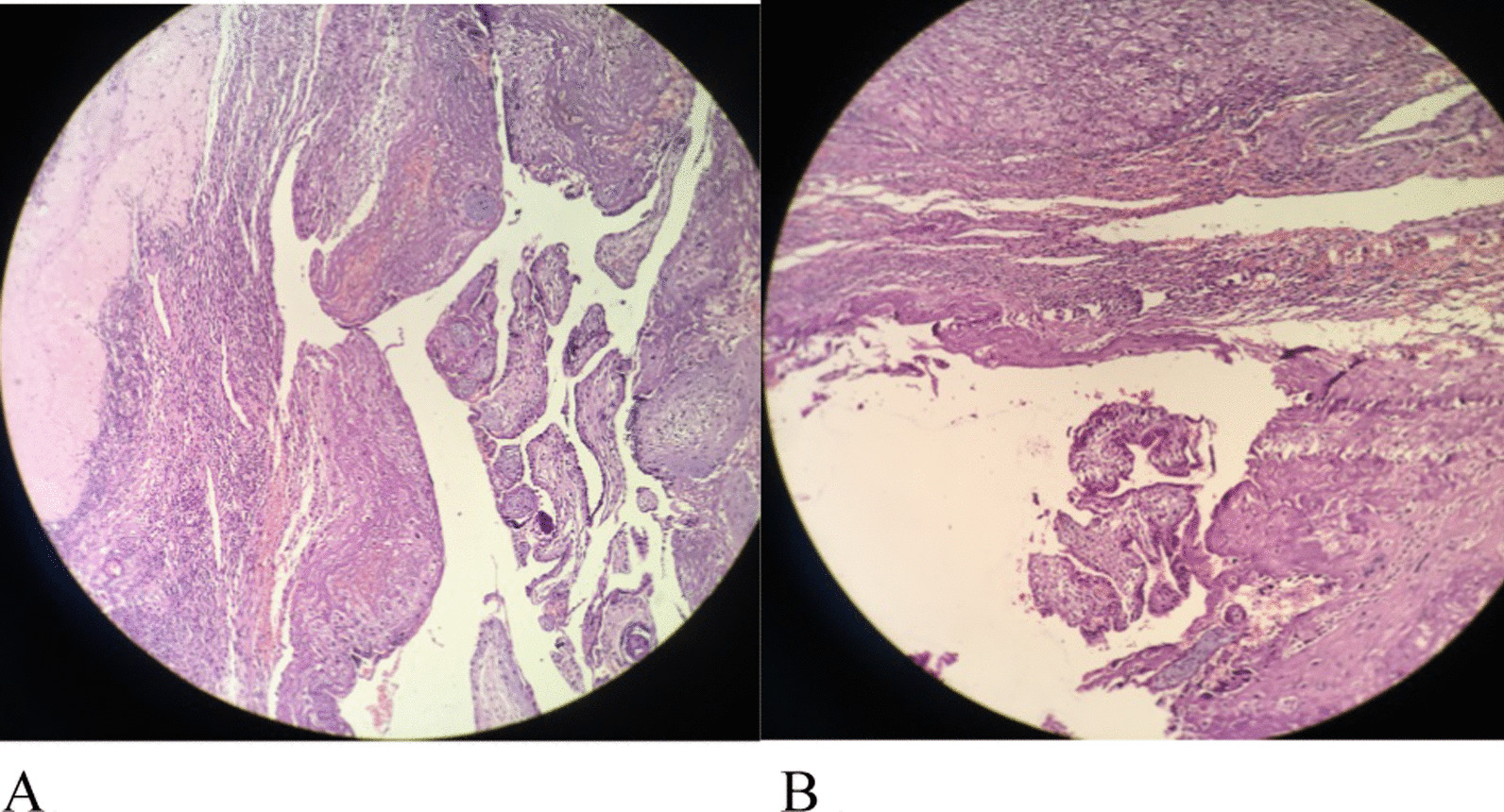
Histopathological examination of the samples. **A** Corpus albicans is in the sack wall. **B** Luteinized follicular cyst is in the sack wall

The patient was diagnosed with OEP and underwent laparotomy surgery, which revealed a gestational sac in the left ovary with visible FHR and about 100 mL of blood, which was evacuated. The gestational sac was surgically removed, and a partial left salpingo-oophorectomy was performed. Concerning the patient's gravidity and age, the patient's left fallopian tube was completely resected for ovarian cancer prophylaxis [7]. We resected the left ovary partially to remove the gestational sac, which was entirely in the left ovary. A live fetus was in the gestational sac at 12 weeks gestational age (Fig. 2). Her post-op β-HCG level was 1901. She was discharged in a stable condition 2 days postoperatively. The histopathological examination of the samples confirmed the diagnosis of OEP (Fig. 3). In the histopathological examination, the tube was intact and clearly separated from the ovary. In addition, ovarian tissue was present in the sack wall (Fig. 3). The patient was followed for 1 month; β-HCG decreased gradually, with no complications in this period.

## Discussion

In the past few years, ectopic pregnancy rates have increased in Iran from 1.9 to 3.7 per 1000 pregnancies [8]. The incidence of OEP has also increased over the past decades, from 0.7% to 1% of all ectopic pregnancies in the 1950s to up to 3.5% in recent studies [9, 10]. Although there is no known definite association, the incidence of OEP is reported to be higher among IUD users [11], which was the case in the reported patient. The patient’s parity was 3, and there are controversies in studies on the association between parity and risk of ectopic pregnancy. While some studies have reported a higher incidence of ectopic pregnancy in patients with higher parity, others have mentioned otherwise [12–14]. In a study by Ehsan *et al.*, mean parity was 2.66 in patients with ectopic pregnancy, which is similar to our case [15]. The assisted reproductive technique (ART) is another probable risk factor for OEP [16].

In previous studies, the mean gestational age at time of diagnosis of OEP was about 7 weeks in the majority of cases [1, 17]. In this case, the patient was unique in this regard as the OEP had lasted for 12 weeks. Different institutes have reported similar OEPs in the first trimester of pregnancy, especially in the earlier stages. Ghasemi Tehran *et al.* reported a patient with a ruptured OEP who had presented with severe abdominal pain at the end of the second month of pregnancy, and the patient was treated by wedge resection of the ovary [18]. Birge *et al.* reported another case of OEP that presented with abdominal pain and vaginal bleeding in the 6th week of pregnancy, which was treated with methotrexate [19]. There are also studies reporting OEPs lasting full-term pregnancy [20, 21]. Huang *et al.* reported a woman in the 36th week of pregnancy diagnosed with OEP and who gave birth to the infant following a laparotomy [20]. Sehgal *et al.* reported the finding of an OEP during a planned cesarean section, which was undiagnosed until that time [21]. These cases indicate the potential for OEP to grow until the later stages of pregnancy, leading to devastating outcomes if rupturing in the late stages. The patient in our study presented with symptoms of abdominal pain and vaginal bleeding, which are the most common in patients with OEP. However, there is still a need for high suspicion to diagnose these patients considering the low prevalence of OEP, especially in the later stages of pregnancy, and unspecific symptoms. Our patient sought medical care immediately after developing symptoms, which led to timely diagnosis. In this case report, the timely diagnosis was key for proper surgical intervention at the right time, and successful management of the patient.

In our patient, β-HCG levels were elevated and TVS revealed the site of the fetus, which were critical for timely diagnosis and intervention. However, diagnostic assessments, such as ultrasound, may not always be indicative. Lee *et al.* reported a patient with OEP who presented with decreased fetal movements in the 38th week of gestation. Ultrasound was not able to detect the OEP in this case and showed a fetus in a vertex position, but a gestational sac was discovered in the left ovary and the definite diagnosis was made intraoperatively [22]. Even though ultrasound has an essential role in diagnosing EP, a high level of suspicion is still needed, as it may fail to diagnose EP in some cases. Routine prenatal assessments may help diagnose EPs in earlier stages and improve outcomes [23]; however, some patients may remain undiagnosed despite routine care [24], adding to the complexity of diagnosis.

Laparoscopic surgery is the preferred intervention for the treatment of patients with OEP. However, as our patient's hemodynamic status was unstable, we performed the laparotomy and resected the fallopian tube entirely, in addition to the gestational sac and surrounding ovarian tissue, reserving the affected ovary by part. As the patient did not have any other pathology, it seemed an appropriate approach, which was successfully done for her [5].

## Conclusion

OEP has the ability to grow until the late stages of pregnancy and may remain asymptomatic or minimally symptomatic even in the late stages. This indicates the importance of prenatal care and careful determination of the fetus site, considering the rise in incidence of EP and OEP. Also, OEP cannot be ruled out in patients of any gestational age, and it should be considered as one of the possible differential diagnoses in females presenting with abdominal pain and vaginal bleeding.

## Data Availability

All related information are reported in this manuscript.
